# The Role of ctDNA in the Management of Non-Small-Cell Lung Cancer in the AI and NGS Era

**DOI:** 10.3390/ijms252413669

**Published:** 2024-12-20

**Authors:** Jacopo Costa, Alexandro Membrino, Carol Zanchetta, Simona Rizzato, Francesco Cortiula, Ciro Rossetto, Giacomo Pelizzari, Giuseppe Aprile, Marianna Macerelli

**Affiliations:** 1 Department of Medicine (DAME), University of Udine, 33100 Udine, Italy; membrino.alexandro@spes.uniud.it (A.M.); zanchetta.carol@spes.uniud.it (C.Z.); 2 Department of Oncology, University Hospital of Udine, 33100 Udine, Italy; simona.rizzato@asufc.sanita.fvg.it (S.R.); francesco.cortiula@asufc.sanita.fvg.it (F.C.); ciro.rossetto@asufc.sanita.fvg.it (C.R.); giacomo.pelizzari@asufc.sanita.fvg.it (G.P.); giuseppe.aprile@asufc.sanita.fvg.it (G.A.); marianna.macerelli@asufc.sanita.fvg.it (M.M.); 3 Department of Respiratory Medicine, Maastricht University Medical Centre, GROW School for Oncology and Reproduction, 6229 ER Maastricht, The Netherlands

**Keywords:** liquid biopsy, ctDNA, NSCLC, NGS, artificial intelligence

## Abstract

Liquid biopsy (LB) involves the analysis of circulating tumour-derived DNA (ctDNA), providing a minimally invasive method for gathering both quantitative and qualitative information. Genomic analysis of ctDNA through next-generation sequencing (NGS) enables comprehensive genetic profiling of tumours, including non-driver alterations that offer prognostic insights. LB can be applied in both early-stage disease settings, for the diagnosis and monitoring of minimal residual disease (MRD), and advanced disease settings, for monitoring treatment response and understanding the mechanisms behind disease progression and tumour heterogeneity. Currently, LB has limited use in clinical practice, primarily due to its significant costs, limited diagnostic yield, and uncertain prognostic role. The application of artificial intelligence (AI) in the medical field is a promising approach to processing extensive information and applying it to individual cases to enhance therapeutic decision-making and refine risk assessment.

## 1. Introduction

A histological diagnosis is essential for the management of patients with non-small-cell lung cancer (NSCLC), along with biomolecular characterization of the disease [1]. Tissue biopsy is the gold standard for reaching a cancer diagnosis, and it should always be pursued [1]. 

**Figure 1:**
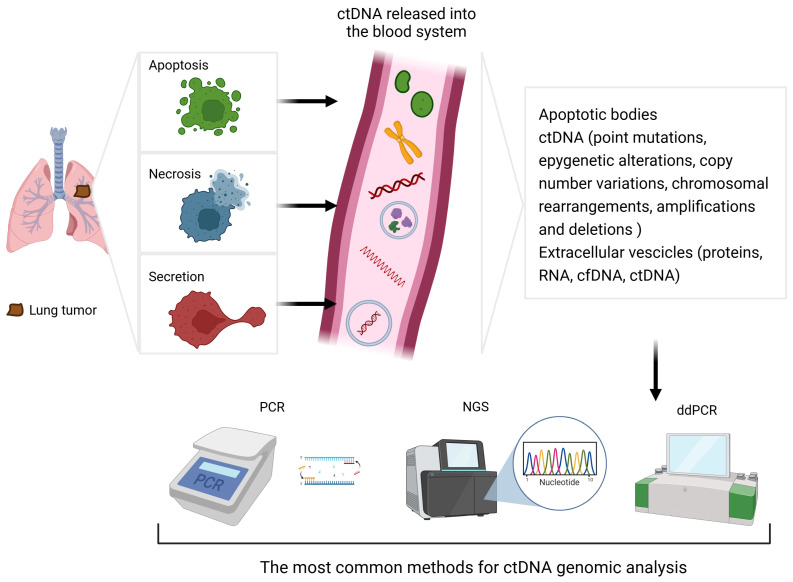
Overview of liquid biopsy and ctDNA genomic analysis. ctDNA = tumour-derived circulating DNA; cfDNA = circulating free DNA; PCR = polymerase chain reaction; NGS = next-generation sequencing; ddPCR = digital droplet polymerase chain reaction.

Liquid biopsy (LB) consists of drawing a blood sample, aiming to harvest circulating tumour cells or tumour-derived circulating DNA (ctDNA) [2,3]. CtDNA fragments are released into the circulation following cellular lysis (necrosis or apoptosis) or through active secretion by neoplastic cells [3]; see Figure 1. The reasons for this active genetic material secretion are not fully understood but may be related to intrinsic signalling mechanisms among tumour cells, potentially promoting distant metastases and intercellular communication [3]. CtDNA might be a valuable surrogate and a complementary test when tissue biopsy is not feasible and when additional material is needed for molecular analysis [4].

In early-stage NSCLC, monitoring minimal residual disease (MRD) may lead to an early diagnosis of recurrence before it becomes radiologically detectable, or it may be useful for defining adjuvant treatment escalation or de-escalation [5]. In advanced NSCLC, ctDNA might be useful for prospective monitoring of the disease and its response to treatments: it can detect disease progression (PD) before standard imaging in patients treated with immune checkpoint inhibitors (ICIs), target therapy, or chemotherapy [6,7]. An early decrease in variant allele frequency (VAF) levels is related to a better prognosis, while an increase or a stable level might indicate no response to therapy [6,7,8,9]. However, an initial increase in ctDNA levels might also indicate a response to therapy due to the disruption of the cell membrane and the shed of ctDNA into the bloodstream [10].

In this narrative review, we present current evidence on the use of liquid biopsy in NSCLC, covering both early-stage and advanced disease, with or without activating genetic alterations (AGAs). Moreover, we present the potential advantages of integrating ctDNA with artificial intelligence and deep learning algorithms.

## 2. Circulating Tumour DNA (ctDNA): Overview, Advantages, and Limitations

### 2.1. Introduction

Circulating free DNA (cfDNA) consists of short double-stranded chains of less than 200 bp and is physiologically present in healthy individuals at very low levels (5–10 ng/mL) [2,3,11]. It is derived mostly from leukocyte turnover, and its concentration can increase in cases of systemic inflammation or pregnancy, but in the presence of neoplastic disease, this increase is exponential. In these cases, cfDNA production exceeds its clearance in filtering structures such as the kidneys, spleen, and lymph nodes. The direct correlation between cfDNA and oncological diseases was first demonstrated in 1977 [11]. The half-life of circulating DNA chains is extremely short, less than 1 h, due to the presence of circulating enzymes [12,13]. Therefore, qualitative and quantitative analyses of ctDNA allow for a real-time snapshot of tumour burden.

The process of ctDNA extraction involves a series of steps to ensure the optimal diagnostic yield. The plasma used must be centrifuged to eliminate leukocytes and prevent contamination of the samples by non-tumour DNA [13]. Blood samples should be stored in a tube containing ethylenediaminetetra-acetic acid (EDTA) or another anticoagulant to avoid white blood cell (WBC) lysis and should ideally be processed within 4 h of their collection [2]. Despite the correct separation of leukocytes, the fraction of ctDNA within cfDNA is generally less than 1%, requiring gene analysis methods with the appropriate sensitivity. The expression “detection limit” refers to the minimum percentage of mutated ctDNA that the method in question can detect within the pool of plasma cfDNA.

Currently, the methods that provide the best results are digital droplet PCR (ddPCR) and next-generation sequencing (NGS). ddPCR is considered the gold standard for the detection of known alterations, while NGS is more suitable for the detection of rare and/or unknown mutations [14]. An overview of the most commonly used techniques is provided in Table 1.

### 2.2. Accuracy

The most important limitation of ctDNA’s implementation in clinical practice is its accuracy. TC-guided biopsy has sensitivity of 92.1% and a specificity approaching 100%, while flexible bronchoscopy has a sensitivity of 88% for hilar lesions and up to 78% for more peripheral tumours [15,16]. On the other side, for liquid biopsy, various significant meta-analyses published over the past decade have demonstrated a sensitivity in the detection of *EGFR* mutations ranging from 59 to 70% and a specificity between 80 and 98% [17,18,19,20,21,22,23,24,25]. The results of these meta-analyses are summarized in Table 2.

One of the first studies to test ctDNA in NSCLC patients was the IFUM, which investigated, as part of the exploratory biomarker objectives, the role of ctDNA in the diagnostic phase. A total of 652 pairs of biopsy and plasma samples were analysed in a single central laboratory, revealing a concordance of 94.3%, with PCR-based ctDNA sensitivity in *EGFR* mutation detection of 65.7% and a specificity of 99.8% (PPV: 98.6%; NPV: 93.8%) [2,26].

The ASSESS study was one of the first to evaluate the use of ctDNA in a real-world and multicentre setting, assessing the variability in ctDNA’s accuracy for *EGFR* mutation detection based on the techniques used in peripheral laboratories, including both PCR- and sequencing-based methods. The biopsy/plasma concordance was 89%, and the overall sensitivity did not reach 50%. This finding underscores the need for highly sensitive methods and standardised techniques across all laboratories [27].

Papadimitrakopoulou et al., utilising large datasets from the AURA3 study, assessed the concordance between 562 pairs of biopsy and plasma samples and the accuracy of ctDNA in detecting *EGFR* mutations (Ex19del, L858R, and T790M I/II generation TKI resistance mutation) using PCR, ddPCR, and NGS [28]. For common mutations, the sensitivity was comparable across all three techniques (Ex19del: 84%, 73%, and 79%; L858R: 60%, 70%, and 63%), while for detecting T790M and uncommon or rare mutations, NGS’s reliability was higher compared to that of PCR-based methods.

The meta-analyses and studies cited employed various analytical techniques, reflecting the historical context in which the studies were conducted, including both PCR-based and NGS-based methods. The accuracy of ctDNA, in terms of its sensitivity and specificity, appears to be higher when an NGS-based technique is used. NGS’s sensitivity ranges from 62 to 87% compared to 59 to 70% in the pooled meta-analyses, while NGS’s specificity ranges from 89 to 98% versus 80 to 98% [17,18,19,20,21,22,23,24,25].

Another factor that influences the sensitivity of LB is the tumour burden, particularly the presence of metastases in the liver, bones, and lymph nodes [23]; in these cases, the probability of detecting an *EGFR* mutation is higher, as reported by Dal Maso et al. (OR: 3.95–8.62). The same study highlighted that the presence of clinical and/or radiological disease progression was another factor determining the sensitivity of LB [29].

So, LB harbours a 30–40% risk of false negative results, potentially limiting its use as an independent diagnostic or monitoring method in clinical practice. Its results must be interpreted alongside a patient’s overall clinical situation, radiologic information, and disease history. Practice-changing randomised clinical trials (RCTs) to determine the optimal use of LB in nearly every disease setting are still awaited. As for now, the FDA and EMA have authorised the use of plasma-detected *EGFR* mutations, regardless of histologic confirmation, for *EGFR*-targeted TKI initiation both in a first-line setting (osimertinib, erlotinib, and gefitinib) or in cases of progression on first- or second-generation TKIs with evidence of T790M mutations (osimertinib) [30,31,32,33,34].

### 2.3. Invasiveness

A key advantage of LB is its minimal invasiveness, allowing for diagnostic investigations in unfit patients and frequent repetition of the test to compensate for its low negative predictive value [35]. Endoscopic or surgical biopsies are procedures associated with potentially severe adverse events and may not always be feasible due to a patient’s condition or the tumour’s location; in Europe, up to 15% of patients are not eligible for histological sampling [36]. A tissue re-biopsy at the time of PD is feasible in only 70% of cases [37]. The ability to obtain qualitative and quantitative information non-invasively is therefore a key feature of ctDNA.

### 2.4. Tumour Heterogeneity

Another potential application of LB is the ability to monitor the sub-clonal evolution of a tumour using the frequent reproducibility of LB. Spatial and temporal heterogeneity plays a crucial role in determining the tumour’s response to treatment, and a ctDNA analysis can provide valuable information to clinicians on tailoring the best therapy to the patient [38]. This heterogeneity is present even in the early stages of the disease, where sub-clonal cell populations may be detected within the primary lung tumour itself, as reported by de Bruin et al. [39]. Up to 30% of the alterations within the mutational pool of the lung tumour mass are sub-clonal, meaning that they are not present in all neoplastic cells [39]. LB allows for comprehensive information on the tumour mutational burden (TMB) from a single sample, unlike traditional biopsy. This information can include the emergence of specific treatment-resistant mutations or the coexistence of various alterations [38,39,40].

### 2.5. Cost

One of the major drawbacks of the LB approach is its cost. The price of a ctDNA NGS analysis is approximately EUR 600–800, with most of this expense attributed to the consumables included in the single-use kit [41,42]. The use of non-proprietary liquid biopsy NGS panels can significantly reduce the costs (e.g., Guardant360™ being CAD 4447 vs. a non-proprietary kit being CAD 1230) [41]. Similarly, ddPCR kits for monitoring NSCLC patients with known driver mutations are more affordable, with a cost of approximately CAD 375 per test for T790M detection [43]. Performing serial LBs without standard protocols from phase 3 RCTs may lead to unaffordable costs. Some “way-defining” trials are investigating the incremental costs of implementing ctDNA in clinical practice. The VALUE study is one of the first to highlight the cost-effectiveness of LB, comparing the use of LB combined with tissue biopsy (TB) to TB alone in a cohort of 150 treatment-naïve NSCLC patients [44]. This combined approach resulted in an average saving of CAD 3065, largely due to the higher detection rate of actionable mutations through LB, which reduced the drug acquisition costs (targeted therapy was less expensive than chemo-immunotherapy).

## 3. The Role of NGS in Molecular Analyses of ctDNA

### 3.1. Introduction

The use of NGS can increase the sensitivity of ctDNA-based diagnostic methods, and the FDA and EMA have approved two ctDNA-based NGS assays for clinical use: Guardant360 CDx (55 genes) and FoundationOne CDx (311 genes) [45,46]. NGS allows for the analysis of large datasets and provides highly informative qualitative data. It can detect unexpected single-nucleotide variations (SNVs), insertions/deletions, amplifications, fusions, methylations, and microsatellite status. Along with the qualitative data, NGS offers semi-quantitative analysis by calculating the variant allele frequency (VAF), the ratio of mutated DNA copies/alleles to the total circulating DNA/alleles, for all the alterations found. The sensitivity of NGS is higher, compared to that of ddPCR, especially for lower VAFs [47,48]. Highly sensitive techniques and optimal plasma sample processing are essential to minimise contamination and ensure accurate results. The main techniques used to optimise NGS diagnostics are reported in Table 3.

### 3.2. Clonal Haematopoiesis

Tumour-derived circulating DNA is present at much lower concentrations than cfDNA, which primarily originates from the leukocytes. Alterations with a higher VAF are linked to clonal haematopoiesis (CH), a physiological occurrence of gene mutations in highly proliferative leukocyte stem cells [52]. The presence of these alterations is typically related to ageing or the administration of cytotoxic drugs. The genes mainly involved are epigenetic modulators such as *DNMT3A*, *TET2*, *ASXL1*, and *JAK*, with the VAFs often between 10% and 20% [47,48]. These mutations are not pro-oncogenic, unless they are specific alterations related to haematologic diseases. However, when interpreting NGS reports, it is essential to distinguish tumour origin alterations from CH mutations. CH mutations may also affect known oncogenes such as *KRAS*, *PIK3CA*, and *EGFR* in about 10% of cases, complicating NGS analysis [53]. Another example is the detection of *TP53* tumour suppressor gene alterations. Their presence in tumour clones represent a negative predictive factor for the response to TKIs and a negative prognostic factor [35,54]. The same *TP53* alterations are frequently found in haematopoietic stem cells (VAF: 5–15%), making it difficult to distinguish between tumour and haematopoietic origin [52].

### 3.3. NGS’s Reliability and Diagnostic Challenges

Regarding the increasingly widespread implementation of NGS in laboratories worldwide, the FDA launched a monitoring project called Sequencing Quality Control Phase 2 (SEQC2) [55]. Laboratories from four different continents were enrolled to analyse the differences in diagnostic accuracy both across various NGS assays and between laboratories using standardised samples. No significant differences in sensitivity were observed for known alterations with intermediate and high VAFs (0.5–5% and >5%, respectively), achieving an excellent diagnostic performance (sensitivity: 96–100%). The highest rate of false negatives (FNs) was reported for mutations with VAFs < 0.5%, particularly in cases with a low ctDNA input at the start of the amplification process (<10 ng). Reproducibility followed a similar trend, with high concordance for alterations with VAFs > 0.5% (95–100%) and reduced concordance for VAFs between 0.1 and 0.5% (58–83%). Finally, when the same NGS technique was used, no statistically significant differences were observed in the results across the participating laboratories. This analysis highlights that currently, NGS analysis of ctDNA is an excellent diagnostic tool for known alterations with VAFs of at least 0.5%, showing a diagnostic performance proportional to the VAF and good reproducibility across centres. However, sensitivity and reproducibility remain a diagnostic challenge for alterations with very low allele frequencies (<0.5%, particularly <0.1%), requiring further advancements in sequencing technologies.

## 4. Prognostic and Predictive Value of ctDNA: A Brief Overview

### 4.1. Screening

The aim of a screening campaign is to reduce a population’s mortality or improve other clinically significant outcomes due to a specific cause [56]. A screening tool should be non-invasive to improve people adherence, so LB might represent an ideal screening test. However, it is difficult to foresee LB as an effective form of screening due to its low negative predictive value (NPV) [2,3,47]. Its sensitivity is especially low in the early stages of the disease, which is the focus of screening procedures. Solely intra-thoracic localisation of the disease and a low tumour burden are both factors associated with reduced tumour shedding, resulting in a higher rate of false negative results [57]. Moreover, the risk of false negatives is influenced by the pre-test probability: the lower the pre-test probability, the higher the risk of false negatives [58]. Thus, in a screening population, the rate of false negatives will be higher compared to a cohort of patients who have already an NSCLC diagnosis. Another significant limitation is the current societal costs of performing an NGS analysis as a screening procedure [59].

### 4.2. Early-Stage Disease

Minimal residual disease (MRD) is defined as the microscopic remnants of a tumour, primarily consisting of single cells, that persist after radical treatment without any evidence of macroscopic disease. Common imaging techniques cannot detect MRD, while NGS-based LB can identify ctDNA or circulating tumour cells confirming the persistence of a tumour after radical treatment [5]. LB might help to identify patients who may benefit from adjuvant therapy or those who are considered to have been cured. Identifying these populations can avoid unnecessary treatment (and AEs) or can offer the possibility for early treatment adaptation [4,58,60].

Chabon et al., in a cohort of 85 stage I-III NSCLC patients who had undergone radical surgery, demonstrated that LB’s sensitivity was correlated with disease stage (42% in stage I, 67% in stage II, and 88% in stage III), and regardless of the disease extent, patients with detectable preoperative ctDNA had a higher recurrence risk [61,62]. Similar results were found in the LUNGCA-1 trial, where the presence of ctDNA 3 days and/or 1 month after surgery was a strong predictor of recurrence-free survival (RFS) (HR 11.1). Adjuvant therapies were identified as an independent factor for RFS in MRD-positive patients (*p* = 0.002) but not in MRD-negative patients (*p* = 0.283) [63]. Another confirmation came from Gale et al., who emphasised the correlation between ctDNA detection 2–4 weeks after surgery and poorer outcomes regarding RFS (HR = 14.8) and overall survival (OS) (HR = 5.5) [64]. Furthermore, it was observed that ctDNA clearance after surgery in patients with pre-surgery positivity appeared to be significantly associated with an improved RFS (HR for patients without ctDNA clearance = 18.2) [64].

Peng at al. highlighted the predictive role of ctDNA in identifying a higher recurrence risk and allowing for an earlier diagnosis of disease recurrence compared to traditional CT scans (12.6 months) [65]. The role of LB in the anticipation of disease recurrence was also confirmed by Chaudhuri et al., as disease progression was anticipated in 72% of patients by a median of 5.2 months [66]. An overview of the trials mentioned is provided in Table 4.

At ESMO 2024, updated results from two trials were presented regarding perioperative chemo-immunotherapy in patients with resectable NSCLC. Specifically, data were provided on ctDNA molecular response and its prognostic role.

The CheckMate 77T study (N = 461), conducted in a cohort of patients with resectable NSCLC in stages IIA-IIIB, demonstrated the superiority of neoadjuvant chemotherapy combined with nivolumab versus CT alone, followed by one year of adjuvant nivolumab [67]. This benefit was shown in terms of both the median event-free survival (EFS) (40.1 vs. 17.0 months; HR = 0.59) and 24-month EFS rate (65% vs. 44%). An exploratory ctDNA analysis showed that during the neoadjuvant phase, the nivolumab arm achieved a higher ctDNA clearance rate (66% vs. 38%), and this clearance was associated with a higher pCR rate: 50% in patients with ctDNA clearance vs. 0% in those without (overall pCR rate of 25.3%) [68].

The AEGEAN study (N = 802), with a similar design, showed the superiority of durvalumab over a placebo in terms of EFS (NR vs. 25.9 months, HR = 0.68) and the 24-month EFS rate (63.3% vs. 52.4%) [69]. ctDNA clearance during neoadjuvant treatment was similarly correlated with a higher pCR rate (50.0% vs. 14.3% in the experimental arm). Additionally, among initially ctDNA-positive patients, all cases with pCR and 93% of cases with a major pathologic response (MPR) experienced ctDNA clearance by C4D1 [70].

Despite the emerging evidence, the implementation of ctDNA in early-stage disease is currently limited due to the high levels of discordance between plasma ctDNA levels and actual tumour presence [58,61,62]. Consequently, given the high rate of false negatives (15–61%) and the costs required to attain sufficient test sensitivity (with MRD reaching VAFs as low as 0.01% in approximately 50% of patients with disease recurrence), LB has not yet been implemented in clinical practice as a follow-up tool in early-stage NSCLC [57,71,72,73].

### 4.3. Advanced NSCLC

As stated by the NCCN and ESMO NSCLC guidelines, the best approach to determining a tumour’s molecular profile should be an NGS-based analysis, either in tissue or liquid biopsy [74,75]. The treatment of advanced NSCLC is based on the detection of different AGAs, such as epidermal growth factor receptor (*EGFR*), anaplastic lymphoma kinase (*ALK*), c-ros oncogene 1 (*ROS1*), rearranged during transfection (*RET*), Kirsten rat sarcoma virus (*KRAS*), B-Raf proto-oncogene (*BRAF*), and mesenchymal–epithelial transition factor receptor (*MET*) genes [72]. Currently, fusions (such as *ALK* and *ROS-1*) can also be tested through NGS [56,76]. However, it is not always feasible to generate a genomic profile from tissue. Specifically, in Europe, tissue biopsy might not be adequate for molecular analyses and is not feasible in 16,000 patients per year [36]. It has been shown that liquid biopsy can provide genomic information in about 20% of cases when this is not feasible using tissue [77].

The NILE clinical trial evaluated the clinical utility of plasma-based NGS for first-line genotyping in patients with metastatic NSCLC as compared with tissue genotyping, showing that NGS (using the Guardant360™ panel) was not inferior to tissue biopsy in detecting activating genetic alterations (AGAs) [78]. The time to treatment (TtT) initiation was significantly shorter in the ctDNA-driven cohort (18 d versus 31 d) [78]. Leighl et al. demonstrated that combining tissue-based genotyping with NGS allowed for the identification of a larger number of driver mutations, with the added benefit of obtaining results sooner using NGS (9 days vs. 15 days) [79]. Thompson et al., in their work on non-squamous NSCLC, highlighted that ctDNA NGS analysis enables clinicians to provide specific treatment recommendations for patients before the first visit more frequently than traditional tissue NGS analysis (74% vs. 46%). The average time gained was estimated to be 8 days [80]. LB analysis allowed for an earlier initiation of systemic treatment, compared with traditional tissue analysis, even in the LIBELULE study (29 days vs. 39 days) and in the ACCELERATE trial (39 days vs. 62 days) [81,82]. This so-called “plasma-first” approach allows for the early detection of driver mutations, eluding the heterogeneity of tissue sample genotyping, and the anticipation of systemic treatment initiation.

Similar to early-stage NSCLC, in advanced NSCLC, the detection of baseline ctDNA and its monitoring during therapy may help identify patients at higher risk. One of the earliest trials evaluating the role of LB was FASTACT-2, a phase 3 RCT in which NSCLC patients were randomised to receive first-line chemotherapy with either erlotinib or a placebo [83]. In the experimental arm, the ctDNA-based analysis demonstrated a benefit in terms of PFS for patients with a negative baseline LB (13.1 vs. 6.2 months) and for those achieving ctDNA clearance after initial positivity (12.0 vs. 7.2 months) [8]. In the ALEX trial, a phase 3 RCT comparing first-line alectinib and crizotinib in *ALK*-positive NSCLC patients, elevated ctDNA levels in both treatment arms were associated with a reduced PFS (HR = 2.04 for alectinib and 1.83 for crizotinib) and a lower probability of survival (HR = 2.52 and 2.63, respectively) [76,84]. The median ctDNA concentration served as the cut-off, though benefits were observed across various thresholds [76]. Regarding third-generation *EGFR*-TKIs, osimertinib was compared with platinum-based chemotherapy as a second-line therapy in AURA3 and with gefitinib or erlotinib as a first-line treatment in FLAURA [30,85]. The biomarker analysis in AURA3 showed that non-detectable baseline levels of *EGFR*-mutated ctDNA were linked to a longer PFS (mPFS in both arms: 12.4 vs. 6.7 months; HR = 0.48) and OS (mOS in both arms: 44.9 vs. 21.6 months; HR = 0.40) [86]. Similar results were observed in FLAURA, with a benefit in terms of PFS (mPFS in both arms: 19.1 vs. 11.1 months; HR = 0.54) and OS (mOS in both arms: not reached vs. 29.7 months; HR = 0.34) [86]. In both trials, patients with ctDNA clearance after initial positivity had improved PFS and OS outcomes compared to those with persistent *EGFR*ms.

The same applies to advanced NSCLC without actionable mutations: in the BR.36 trial, ctDNA molecular response was used to escalate therapy in patients receiving first-line immunotherapy, adding chemotherapy to the treatment regimen in cases of ctDNA persistence after 6 weeks. ctDNA clearance was associated with a longer PFS (5.0 vs. 2.6 months; HR  =  0.55) and OS (NR vs. 7.2 months; HR  =  0.16) [87]. Pellini et al. showed that the ctDNA clearance after three cycles of chemo-immunotherapy was associated, compared to persistent ctDNA positivity, with a higher ORR (78 versus 43%), a longer mPFS (8.8 months vs. 3.5 months; HR = 0.32), and an extended mOS (NR vs. 8.9 months; HR = 0.22 [88]. In a cohort of patients treated with nivolumab as their second-line treatment or later, Giroux Leprieur et al. found that an increase in ctDNA at the time of the first instrumental reassessment was associated with a remarkable decrease in PFS (0.7 months vs. 12.0 months) and OS (NR vs. 2.1 months) [9].

Identifying patients at greater risk might be useful in clinical practice when more treatment options are available (e.g., first-line treatment for *EGFR*-positive NSCLC), and tailoring anticancer treatment based on ctDNA would avoid unnecessary toxicity for patients, as well as unnecessary societal toxicities. RCTs guided by ctDNA are necessary before implementing a liquid biopsy in clinical practice to optimise the treatment escalation or de-escalation based on tumour molecular response.

LB can also be used in advanced NSCLC to detect the emergence of resistance mutations. The occurrence of specific resistance mutations affects up to 46% of patients treated with third-generation *EGFR* tyrosine kinase inhibitors. The most common mutations involve *MET*, *EGFR*, *PIK3CA*, *ERRB2*, *KRAS*, and *RB1*, with co-occurrence reported in 5–15% of cases [38]. The ELIOS trial (N = 154) utilised an NGS-based ctDNA analysis to evaluate resistance mutations to third-generation *EGFR*-TKIs, comparing baseline liquid biopsies with those taken at the time of radiologic progression. The most common acquired alterations were *MET* amplification (17%) and the *EGFR* C797S mutation (15%) [89]. Berko et al., in a cohort of 41 patients treated with *ALK* TKIs, studied the emergence of resistance mutations through a ctDNA NGS-based analysis. They found that the most frequent “off-target” mutations occurred in the *RAS/PI3K* pathway, alongside acquired secondary mutations in the *ALK* gene [90]. *ALK* mutations such as D1203N/L1196M, G1202R/L1196M, and G1202R/F1174C are recognised as primary or acquired lorlatinib resistance mutations and are more commonly found in patients with concurrent *TP53* mutations [90,91].

The detection of an increasing TMB, marked by relatively low-frequency nonspecific mutations, may indicate a negative prognostic factor for patients with NSCLC. Liu et al. demonstrated that the presence of mutations with a low variant allele frequency (VAF) compared to the maximum somatic allele frequency (MSAF) of the driver mutation is detrimental to overall survival (HR: 1.52; 95% CI: 1.27–1.83; *p* < 0.001) [40]. The VAF/MSAF cut-off was set at 10%.

## 5. Liquid Biopsy and Artificial Intelligence

### 5.1. Definition

In the last 70 years, Information Technology has made progress in leaps and bounds, and the term “Artificial Intelligence” (AI) has become part of common language. Artificial intelligence is an umbrella term for a series of computational strategies reproducing critical human thinking and problem-solving through algorithms for the integration of data and models into all scientific fields. In defining AI, we can describe an overarching system in which machine learning (ML) is one of AI’s subfields that uses self-learning algorithms that derive knowledge from data in order to predict outcomes, while deep learning (DL) is a subset of machine learning that enables us to automate certain processes using a large dataset, eliminating the need for human intervention [92].

**Figure 2:**
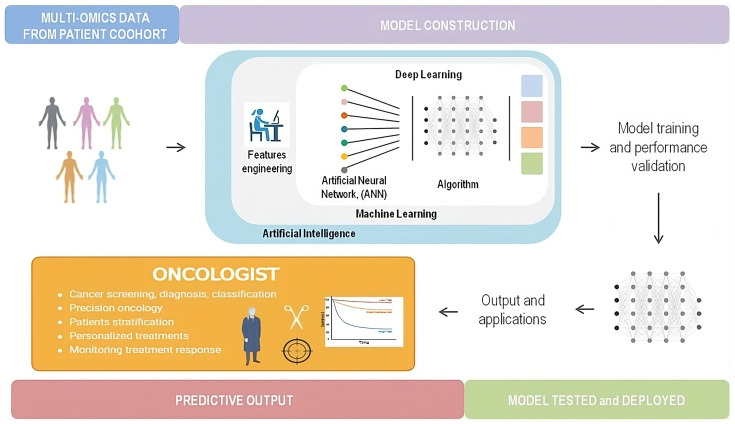
A simple graph to explain the AI workflow from multi-omics data to output and applications. Deep learning is a subfield of machine learning, and machine learning is as a subfield of AI. All algorithms and models should be tested and validated before they are used in clinical trials. Oncologists can apply data derived from AI analyses for a large number of applications, as listed in the graph.

Oncology is one of the most fascinating compasses in which AI can make a difference for managing cancer patients. Currently, the applications of AI range over precision oncology with integration between omics analysis and deep-learning strategies, to cancer diagnosis, screening or classification until the assessment of predictive or prognostic biomarkers other than a possible real-time monitoring of cancer responses [92,93] (Figure 2).

Radiomics and pathomics models are the first scope of AI’s application in oncology, and to date, the FDA has already approved 71 AI-associated devices; more than 80% of these are involved in the area of cancer diagnostics, mostly in the breast cancer field (31%) followed by lung cancer (8.5%), colorectal cancer (7%), and brain tumours (2.8%) [94].

### 5.2. Integration of Artificial Intelligence into ctDNA Analysis: Advancing Precision Oncology

**Figure 3:**
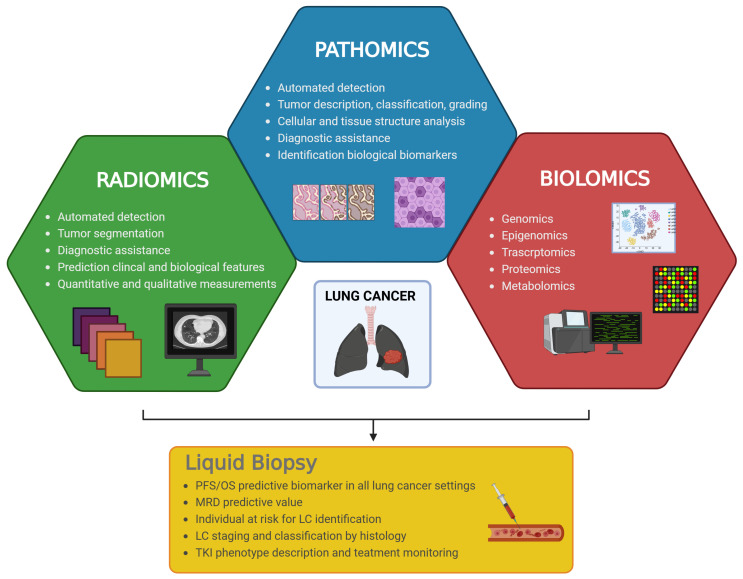
Possible applications in multi-omics fields to dissecting lung cancer and all of the relative diagnostic, prognostic, and therapeutic implications. Artificial intelligence (AI) might support radiologists, pathologists, molecular pathologists, and oncologists, but also surgeons and radiotherapists, in the near future. Liquid biopsy is one fascinating scope for using AI. PFS = progression-free survival; OS = overall survival; MRD = minimal residual disease; LC = lung cancer; TKI = tyrosine kinase inhibitor.

New challenges in AI-enhanced approaches reflect the oncological need to identify prognostic and predictive biomarkers, either for early cancer detection or monitoring treatment responses/survival outcomes (Figure 3). Integration between AI technology and ctDNA analysis is the intriguing next generation, and here, we describe the first results from large phase II and III trials and small case series.

#### 5.2.1. Screening and Risk Assessment

AI-enhanced tools might revolutionise oncological screening by detecting lung cancer at the early stages in healthy individuals and high-risk populations.

Bahado-Singh et al. studied AI platform assays for detecting methylated ctDNA identifying potential biomarkers and defining the pathogenesis of lung cancer [95]. They used an Illumina^®^ array analysis and exploited six AI platforms, both human-supervised (support vector machine (SVM)) and unsupervised machine learning algorithms (deep learning (DL)) to identify cytosine methylation genes across the genome of lung cancer. Data optimisation was performed through a 10-fold cross validation method, and the accuracy of the estimates was guaranteed with a bootstrapping method using random sampling with replacement. The matching results between 10 NSCLC cases and 20 controls identified NSCLC cases with great accuracy (AUC = 1.00; 95% CI: 0.95–1.00), sensitivity, and specificity (with both reaching 100%). Many epigenetically altered genes are known in lung cancer, and AI approaches could integrate clinical, pathological, and dynamic plasma data to diagnose neoplasms through liquid biopsy.

Kim et al. conducted a similar analysis using a deep learning model to identify people with lung cancer from healthy individuals [96]. They developed methylated DNA sequencing and genome-wide Enzymatic Methyl-seq (EM-seq) from lung cancer tissue and from plasma to identify methylated genes, as collected in the Cancer Genome Atlas and Gene Expression Omnibus databases. They developed a convolutional neural network (CNN), a self-propagating neural network based on a hyperparameter tuning method, to distinguish between healthy individuals and patients with NSCLC. They examined 142 NSCLC samples and 56 healthy plasma samples using EM-seq, achieving high accuracy (81.5%; AUC: 0.87) in identifying NSCLC patients.

Mathios et al. studied a machine learning model for detecting tumour-derived ctDNA in a large cohort of patients with a high risk of developing lung cancer [97]. They developed a genome-wide system for the analysis of cfDNA fragmentation profiles called DELFI (DNA evaluation of fragments for early interception), and they validated this methodology in a large cohort of 385 consecutive smoker patients with a suspected pulmonary nodule (323 patients had dyspnoea or a cough). Using data from a cohort of 385 healthy individuals and 46 LC patients, they developed a reproducible predictive model for early- and late-stage cancer detection. Clinical, laboratory, and radiological features were used to train the machine learning algorithm, which was validated through a fivefold cross-validation process with a control fold to evaluate the model’s performance. They demonstrated that a non-invasive approach, using DL applied to ctDNA, could provide a valuable opportunity for cancer detection across different stages with high accuracy (AUC = 0.98) and sensitivity (94%), while also classifying lung cancer by histology (NSCLC vs. SCLC).

Shin et al. used a machine learning algorithm to detect ctDNA in patients with chronic obstructive pulmonary disease (COPD) to identify groups at high risk of developing lung cancer [98]. They analysed, using deep sequencing techniques, ctDNA from 177 patients to identify prespecified mutations based on the allele frequencies. The authors employed four ML models to integrate clinical characteristics, including the emphysema index, laboratory findings, chest computed tomography data, and ctDNA assessments. Leave-one-out cross-validation (LOOCV) was used to train the model, selecting variables with a significant association with the presence of ctDNA mutations (*p* < 0.01) in a logistic regression (LR) algorithm, a supervised ML model. The detection of at least one notable ctDNA mutation (found in 54 patients, 30.5%) was associated with an increased risk of lung cancer development, a more advanced radiological and clinical stage at diagnosis, and a higher prevalence of SCLC compared to patients without a ctDNA detection (*p* = 0.01). During the follow-up, 51 patients (28.8%) died due to lung cancer, and a higher proportion of them had a ctDNA detection during the follow-up period (51.9% vs. 18.7%, *p* < 0.001).

#### 5.2.2. Early-Stage and MRD Monitoring

Widman et al. addressed the challenges of the low sensitivity and high false negative rates in MRD detection by developing MRD-EDGE, an ultra-sensitive machine-learning-guided ctDNA analysis method using whole-genome sequencing (WGS) [99]. They utilised feedforward semi-supervised neural networks, including a regional multilayer perceptron (MLP) and a fragment convolutional neural network (CNN), to create a deep learning model based on the detection of single-nucleotide variants (SNVs) and copy number variants (CNVs) in ctDNA from patients with melanoma and colorectal and lung cancer.

In the post-operative setting, MRD-EDGE was used to analyse plasma samples from 15 stage III colorectal cancer (CRC) patients and 40 healthy individuals. The presence of post-adjuvant MRD correlated with a shorter DFS, while no recurrence was recorded from patients without a ctDNA detection [99].

In the neoadjuvant setting, they tested MRD-EDGE in a phase II trial in which 22 early-stage NSCLC patients had been treated with durvalumab with or without stereotactic radiation before surgery [100]. MRD-EDGE was highly sensitive for pretreatment ctDNA assessments (AUC: 0.98; 95% CI: 0.95–1.00), and this system allowed them to monitor the ctDNA kinetics: increased ctDNA shedding was identified during stereotactic body radiation therapy (SBRT), with a depth decrease after 4 weeks. There were plasma samples available for 14 patients post-surgery, and MRD detection correlated with a shorter disease-free survival (*p* = 0.036).

MRD-EDGE has also been used to monitor the response to immunotherapy, starting with melanoma patients [99]. By analysing the ctDNA kinetics in 37 patients, with samples collected at baseline and 3 weeks and 6 weeks after treatment initiation, it was found that a decrease in the ctDNA tumour fraction was associated with a longer PFS (*p* = 0.01) and OS (*p* = 0.03). Reduced efficacy of immune checkpoint inhibitor (ICI) therapy was also observed in conjunction with steroid therapy for immune-related adverse events (AEs). These data were later confirmed in patients with SCLC treated with ICI and chemotherapy combinations, where an increase in ctDNA at week 3 correlated with a shorter PFS and OS [99].

#### 5.2.3. Advanced-Stage Treatment Monitoring

In the randomised phase III Impower150 trial, chemo-naïve metastatic NSCLC patients were randomised to receive carboplatin–paclitaxel–bevacizumab with or without atezolizumab [101]. As an exploratory biomarker analysis, ctDNA was collected across five prespecified time points (at baseline and on day 1 of cycles 2, 3, 4, and 8) in 466 enrolled patients. Two AI-based algorithms were developed, incorporating clinical, laboratory, and radiological features.

In the first case, considering the baseline sample and the one taken on day one of cycle 3, the risk classes were identified in relation to RECIST response grade after 6 weeks of treatment. In cases of stable disease (SD), the outcome for patients classified as “high-risk” versus “low–intermediate-risk” was 7.1 months versus 22.3 months (HR = 3.2 (2.0–5.3); *p* < 0.001). This hazard ratio remained consistent even when considering a global partial response (PR) according to RECIST (HR = 3.3 (1.7–6.4), *p* < 0.001). The AI model, based on an artificial neural network, was trained and validated using leave-one-out cross-validation (LOOCV) [102].

In the second analysis, the algorithm was enhanced by incorporating all subsequent ctDNA samples, evaluating their kinetics over time and correlating them with PFS and OS. The biological changes in 17 ctDNA markers (including ctDNA concentration, tumour molecules in the blood, VAFs, and the total number of mutations) over time correlated with PFS and OS, dividing the patients into high-, intermediate-, and low-risk groups. Complete ctDNA clearance by day 1 of cycle 3 or 4 was associated with a long-term benefit (C-index = 0.69), better than the combined ctDNA and radiologic assessment at the 6-week time point [103].

#### 5.2.4. Radiomics

The integration between radiomics and ctDNA is another interesting AI field.

Cucchiara et al. described one of the first attempts to integrate radiomics data and liquid biopsy in a series of patients with *EGFR*-mutant cancer to overcome the heterogeneity of [104] *EGFR* resistance mutations. They used a logistic LASSO (least absolute shrinkage and selection operator) regression model with a 27-fold Monte Carlo cross-validation method to select radiomic features to combine images and *EGFR* mutation types (both activating or resistance mutations). They monitored seven cases of advanced *EGFR*-mutant cancer, combining data from cfDNA analysed using ddPCR and radiological data analysed using LifeX^®^ software (v6.30). This approach was able to predict the *EGFR*-TKI response and resistance during treatment through a computational model.

He et al. combined a deep learning radiomic biomarker approach with TMB data from 327 patients with NSCLC treated with immune checkpoint inhibitors (ICIs) [105]. Through a deep learning model, they built a radiomic biomarker of tumour mutational burden (TMB) to distinguish between a high and low TMB (AUC: 0.85; 95% CI: 0.84 to 0.87). They verified how an AI-enhanced approach, integrated with radiological and clinical features (such as performance status), could predict and monitor the ICI response in patients with advanced lung cancer.

Yousefi and colleagues integrated ctDNA data and clinical and lab features with radiomics phenotypes to predict the *EGFR*-TKI response in advanced lung cancer patients [106]. They used a radiomic workflow where the tumour was segmented in 3D and a two-level hierarchical clustering algorithm to cluster the radiomic signatures into specific and prognostic/predictive phenotypes. They analysed 40 patients with *EGFR*-mutant cancer and identified two radiomic phenotypes. Regarding PFS, the ML model integrated with radiomics had a C-index of 0.77 versus 0.73 and a greater fit than a model without radiomic data (likelihood ratio test *p* = 0.01). The same was found for OS (C-index 0.83 versus 0.80), with a slightly higher LTR (0.08).

## 6. Conclusions

**Figure 4:**
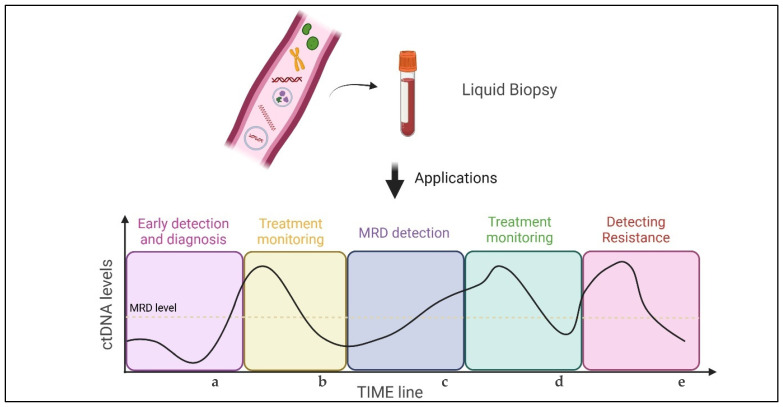
Overview of potential applications of liquid biopsy in NSCLC patient management. (**a**) Diagnosis: Complementary or exclusive use of LB compared to tissue biopsy. (**b**) Early-stage treatment escalation/de-escalation based on early molecular response of ctDNA. (**c**) Monitoring minimal residual disease (MRD) for early detection of disease recurrence. (**d**) Advanced-stage treatment escalation/de-escalation based on ctDNA levels. (**e**) Detecting specific resistance mutations to guide targeted therapies.

Liquid biopsy has demonstrated significant value in managing patients with NSCLC. Its applications are diverse, spanning from diagnosis and follow-up in early-stage disease to monitoring therapeutic response and understanding the mechanisms underlying disease progression and tumour heterogeneity in more advanced stages (Figure 4). Numerous clinical trials are laying the groundwork for future implementations of ctDNA in clinical practice. Currently, however, the limited sensitivity and cost of LB restrict its routine use. At present, LB is used as a complementary or exclusive diagnostic tool in NSCLC patient management. Phase 3 RCTs will be essential to shift clinical practice toward using ctDNA for tumour heterogeneity assessments and tailoring genomic treatment. At the moment, the phase 3 CheckMate 77T and AEGEAN trials, conducted in the perioperative setting, are providing the first insights into the use of ctDNA in clinical practice. The phase 2 TARGET trial, whose primary results are not yet available, will also incorporate ctDNA into MRD monitoring in early-stage *EGFR*-positive disease [107].

The integration of AI-guided techniques may help to mitigate these drawbacks and optimise the diagnostic yield in the future. The field surrounding the implementation of AI in medicine, and particularly in oncology, is vast and experiencing exponential growth. However, the development of new AI-based algorithms must undergo validation both in terms of mathematical and algorithmic rigor and from a methodological perspective within well-constructed clinical trials. Currently, the FDA has approved 71 AI-associated devices, although their clinical applications remain limited.

## Figures and Tables

**Table 1:** Description of the mechanism, advantages, drawbacks, and detection limit of the three most common methods for ctDNA genomic analysis. PCR = polymerase chain reaction; ddPCR = digital droplet polymerase chain reaction; NGS = next-generation sequencing.

Method	Mechanism	Advantages	Drawbacks	Detection Limit
PCR	Non-selective DNA amplification using the polymerase I enzyme	Low cost Ready-to-use kits	Detection of known and/or expected mutations only	0.1–1% [2]
ddPCR	Amplification of DNA segments of interest within thousands of nanolitre-sized droplets	Low cost High sensitivity Quantitative analysis (copies/mL)	Detection of known and/or expected mutations only	0.01–0.1%, up to 0.001% [14]
NGS	Analysis of the DNA sequence by identifying each individual nucleotide in rapid succession	Systematic sequencing Detection of any DNA alteration	High cost Longer time to yield results Variable sensitivity	0.01–2%, based on computational power [2]

**Table 2:** Results from the most significant meta-analyses on the diagnostic accuracy of ctDNA in NSCLC harbouring *EGFR* mutations. PCR = polymerase chain reaction; HRM = high-resolution melting; ddPCR = digital droplet polymerase chain reaction; NGS = next-generation sequencing; NE = not evaluable.

Metanalysis	Year	Technique	Pooled Sensitivity	Pooled Specificity	%NGS	NGS Sensitivity	NGS Specificity
Luo et al. [19]	2014	PCR/HRM	67% (52–80%)	93,5% (89–96%)			
Mao et al. [21]	2015	PCR/NGS	61% (50–71%)	90% (85–94%)	15% (222/14840)	70% (46–87%)	90% (49–99%)
Qiu et al. [20]	2015	PCR/HRM	62% (51–72%)	96% (93–98%)			
Qian et al. [18]	2016	PCR/HRM	60% (57–62%)	94% (93–95%)			
Passiglia et al. [23]	2018	PCR/ddPCR/NGS	67% (64–70%)	80% (77–83%)	10% (169/1639)	87% (76–95%)	89% (82–94%)
Zhou et al. [22]	2020	PCR/ddPCR/NGS	70% (63–75%)	98% (96–99%)	NE	80% (64–96%)	98% (96–100%)
Wang et al. [25]	2021	PCR/HRM7NGS	68% (60–75%)	98% (95–99%)	NE	79% (NE)	98% (NE)
Franzi et al. [24]	2023	PCR/ddPCR/NGS	59% (41–75%)	96% (92–97%)	39% (668/1711)	62% (46–76%)	95% (89–98%)

**Table 3:** Description of the mechanisms and efficiency of the three main techniques used to optimise NGS’s accuracy for ctDNA analysis. UMIs = unique molecular identifiers; TAM-SEQ = tagged-amplicon deep sequencing; CAPP-SEQ = cancer personalised profiling by deep sequencing.

Method	Mechanism	Accuracy
UMIs	Selective analysis of DNA fragments bound to predefined nucleotide sequences	Detection limit: 0.1~0.5% [49]
TAM-SEQ	Selective amplification of DNA fragments using predefined primers	Detection limit: 0.25~2% Sensitivity: 94–97% [50]
CAPP-SEQ	Amplification of selected DNA fragments based on databases and bioinformatics algorithms	Detection limit: down to 0.02% Sensitivity: up to 100% [51]

**Table 4:** Results of trials regarding the use of ctDNA to monitor MRD as a prognostic factor. RFS = relapse-free survival; FFM = freedom from metastasis; OS = overall survival, IVr = resectable stage IV.

Reference	Stage	No. Patients	Pretreatment Positive ctDNA	Post-Treatment Positive ctDNA
Chabon et al. [62]	I–III	85	RFS, HR = 3.4 (*p* = 0.026) FFM, HR = 6.0 (*p* < 0.001)	RFS, HR = 4.5 (*p* < 0.001)
Xia et al. [63]	I–III	427	RFS, HR = 4.2 (*p* < 0.001)	RFS, HR = 11.1 (*p* < 0.001)
Gale et al. [64]	I–III	88	RFS, HR = 3.1 (*p* = 0.003) OS, HR = 3.0 (*p* = 0.01)	RFS, HR = 14.8 (*p* < 0.001) OS, HR = 5.5 (*p* < 0.001)
Peng et al. [65]	I–IVr	77	RFS, HR = 3.6 (*p* < 0.001) OS, HR = 4.8 (*p* = 0.0013)	RFS, HR = 2.9 (*p* = 0.0035) OS, HR = 3.0 (*p* = 0.0086)
Chaudhuri et al. [66]	I–III	40	NE	RFS HR = 16.3 (*p* = 0.001) OS HR = 10.9 (*p* < 0.001)

## Data Availability

No new data were created.
